# Giant Spherical Cluster with *I*-C_140_ Fullerene Topology**

**DOI:** 10.1002/anie.201505516

**Published:** 2015-09-28

**Authors:** Sebastian Heinl, Eugenia Peresypkina, Jörg Sutter, Manfred Scheer

**Affiliations:** Institute of Inorganic Chemistry, University of Regensburg Universitätsstrasse 31, 93040 Regensburg (Germany) E-mail: Manfred.Scheer@ur.de; Nikolaev Institute of Inorganic Chemistry, SB RAS Ak. Lavrentiev prosp. 3, 630090 Novosibirsk (Russia); Department of Chemistry and Pharmacy, Inorganic Chemistry, FAU Erlangen-Nürnberg (FAU) Egerlandstrasse 1, 91058 Erlangen (Germany)

**Keywords:** C_140_ fullerene, Cp^BIG^, giant clusters, pentaphosphaferrocene, supramolecular chemistry

## Abstract

We report on an effective cluster expansion of CuBr-linked aggregates by the increase of the steric bulk of the Cp^R^ ligand in the pentatopic molecules [Cp^R^Fe(η^5^-P_5_)]. Using [Cp^BIG^Fe(η^5^-P_5_)] (Cp^BIG^=C_5_(4-*n*BuC_6_H_4_)_5_), the novel multishell aggregate [{Cp^BIG^Fe(η^5:2:1:1:1:1:1^-P_5_)}_12_(CuBr)_92_] is obtained. It shows topological analogy to the theoretically predicted I-C_140_ fullerene molecule. The spherical cluster was comprehensively characterized by various methods in solution and in the solid state.

Since the discovery of the C_60_ buckminsterfullerene[1] in 1985 by Curl and Smalley, the synthesis of larger fullerenes continues to be the focus of research (Figure 1).[2] Additionally, the structural diversity, functionalization, and usage of fullerenes have been investigated extensively.[3] However, the number of larger fullerenes is still limited. The largest structurally characterized examples is the endohedral fullerene Sm_2_@C_104_ of Balch et al.[4] and the chlorinated compounds C_104_Cl_16_ and C_104_Cl_24_, both reported by Yang et al.[5] Also much larger fullerenes up to C_418_ have been detected by mass spectrometry.[6] Another strategy was pursued by Oshima and Takayanagi et al. who applied bias voltages on an amorphous carbon agglomerate between gold electrodes with a transmission electron microscope/scanning tunneling microscope.[7] By size comparison they estimate that the fullerenes C_140_, C_180_, C_240_, C_260_, and C_620_ were formed. However, the structural identity of all these larger carbon clusters is unclear, since formation of dimers or aggregates (e.g. C_120_⇄(C_60_)_2_),[8] onion-like structures,[9] and fullerene isomers (still fulfilling the isolated pentagon rule)[2, 9] is possible. Generally, it was calculated that the ball-shaped arrangements are energetically preferred over capsule-like structures.[10] In this context, the most stable isomer of C_140_ fullerene was calculated to be icosahedral *I*-C_140_.[11]

**Figure 1:**
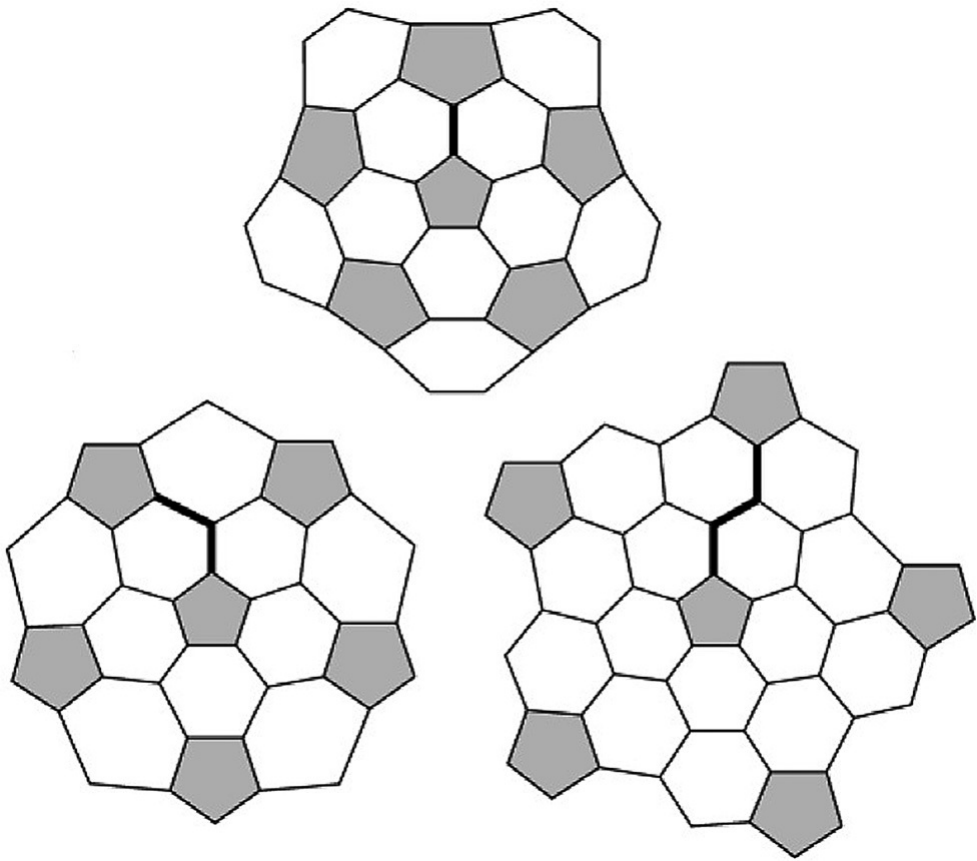
2D projections of sections of the fullerene frameworks of C_60_ (top), *I_h_*-C_80_ (bottom left), and *I*-C_140_ (bottom right). The shortest connections between the C_5_ rings are emphasized in bold.

Carbon is not the only element suitable for the formation of spherical macromolecules. In polyoxometalate chemistry, Keplerates[12] with the simplified formula [{(M^VI^)Mo^VI^_5_}_12_(linker)_30_] (M=Mo, W; linker=Mo_2_O_4_(acetate)^+^, VO_2_^+^, Cr^3+^, Fe^3+^) are well known.[13] In addition, pentasubstituted Cp^R^ derivatives can also form nanoballs in combination with metal salts, as Williams et al. showed for the system [(C_5_H_4_PO*t*Bu_2_)Fe{C_5_(4-pyridyl)_5_}] and Cu^I^ cations.[14] Furthermore, Wright et al. obtained a fullerene-type metal–organic framework with the sodium salt of [C_5_(CN)_5_]^−^.[15] In the field of coordination compounds, Fujita et al. succeeded in the preparation of spherical compounds from oligopodal pyridine linkers and Pd^II^ moieties. However, none of these materials display fullerene-like topology.[16] In contrast, the pentaphosphaferrocenes [Cp^R^Fe(η^5^-P_5_)] were shown to be excellent building blocks in combination with Cu^I^ halides for the formation of fullerene-like supramolecules, for example, [{Cp*Fe(η^5:1:1:1:1:1^-P_5_)}_12_{CuCl}_10_{Cu_2_Cl_3_}_5_{Cu(CH_3_CN)_2_}_5_] (Cp*=C_5_Me_5_),[17] which comprises 90 non-carbon scaffold atoms. Depending on the Cu^I^ halide, the template and the reaction conditions, different structural motifs were obtained following the fullerene topology predetermined by the *cyclo*-P_5_ rings (e.g. scaffolds with a *I_h_*-C_80_ related core, cf. Figure 1).[18] With [Cp^Bn^Fe(η^5^-P_5_)] (Cp^Bn^=C_5_(CH_2_Ph)_5_)[19] as a building block, products with good solubility are obtained.[20]

Almost all the nanosized compounds described above (*d*=2.1–2.8 nm) follow the fullerene topology, containing 12 five-membered rings and (*n*−20)/2 six-membered rings.[18, 20] Therefore the question arises whether the use of building blocks much larger in size results in the formation of same-sized spheres with fewer *cyclo*-P_5_ units or much larger spheres that arise from the self-assembly of 12 pentaphosphaferrocenes. The latter possibility might open the way to structurally unknown relatives of the largest known fullerenes. Therefore the very bulky building block [Cp^BIG^Fe(η^5^-P_5_)] (**1**; Cp^BIG^=C_5_(4-*n*BuC_6_H_4_)_5_) was synthesized,[21] in which the radius of the Cp^BIG^ ligand (*r*≍9 Å) is about three times larger than that of the Cp* ligand (*r*≍3 Å).[22]

Herein we report on the preparation and characterization of the “expanded” cluster [{Cp^BIG^Fe(η^5:2:1:1:1:1:1^-P_5_)}_12_Cu_70_Br_83_] (**2**), which shows the same structural topology as the theoretically predicted *I*-C_140_ fullerene (Figure 1). This Br/Cu/P scaffold is the non-carbon version of the *I*-C_140_ fullerene, and its structural characterization reveals the potential of the building blocks as well as the fullerene building concept in supramolecular chemistry.

Addition of a CH_2_Cl_2_ solution of **1** to a CH_3_CN solution of CuBr results in the formation of the novel giant supramolecule [{Cp^BIG^Fe(η^5:2:1:1:1:1:1^-P_5_)}_12_Cu_70_Br_83_] (**2**).[23] Compound **2** has poor solubility in CH_2_Cl_2_ and is insoluble in hexane, toluene, CH_3_CN, and Et_2_O. By diffusion of toluene into CH_2_Cl_2_ solutions, it crystallizes in the monoclinic space group *C*2/*c* as black cubes and can be isolated in a yield of 25 %, which is astonishing for a compound of this type and size. Single crystals could also be grown when hexane is used instead of toluene. However, in the latter case they quickly lose crystallinity when the crystals are removed from the mother liquor.

The unprecedented scaffold of **2** consists of 92 copper and 92 bromide positions as well as 12 units of **1**. The supramolecule has an outer diameter of about 3.5 nm and shows a three-shell core structure. If the Cp^BIG^ ligands are taken into account even a four-shell structure is present. This is in contrast to the compounds constructed from [Cp*Fe(η^5^-P_5_)], which exclusively show single-shell aggregates with cavities occupied by a template or a solvent molecule.[17–19] The space-filling view of **2** (Figure 2 a) exhibits a tight arrangement of the twelve Cp^BIG^ ligands forming a distorted pentagonal dodecahedron as the outer shell of the giant molecule. The supramolecules are ordered in a slightly distorted cubic close packing (Figure S4), which is typical for the packing of spheres.[24]

**Figure 2:**
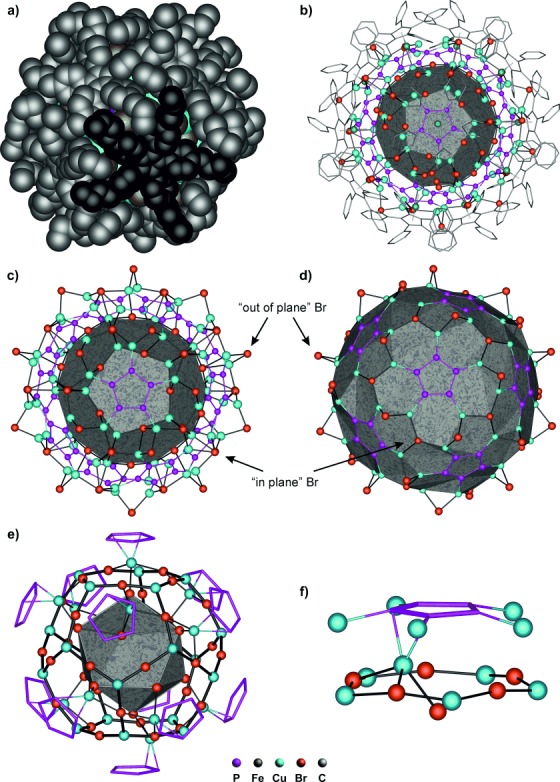
Molecular anatomy of the organometallic cover and the three-shell core structure of 2. a) Entire supramolecule as a space-filling model; one of the Cp^BIG^ ligands is colored in black and H atoms are omitted for clarity. b) Supramolecule as a ball-and-stick model with the inner Cu_20_ dodecahedron; *n*-butyl groups are omitted for clarity. c) Inorganic scaffold of 2 with the inner Cu_20_ dodecahedron. d) Idealized inorganic scaffold of 2 representing *I*-C_140_ analogy. e) Inner CuBr framework including all partly occupied positions of copper and bromine and including the inner Br_12_ icosahedron, which is dual to the CuBr dodecahedron (thick bonds). f) Interconnection of the outer and inner shells via additional η^2^-coordination of Cu ions to 1 (one-twelfth of the supramolecule is shown).

In **2** all P atoms of the pentaphosphaferrocene units **1** are coordinated to Cu ions resulting in a 1,2,3,4,5 coordination mode (Figure 2 b–d). Each Cu ion of the outer shell additionally binds to three bromide ions: one points inward, one is “in-plane”, and one “out-of-plane” (see Figure 2 c). These Cu and Br ions in combination with the P atoms of **1** form the outer shell. Interestingly, with 12 five-membered *cyclo*-P_5_ rings and 60 six-membered P_2_Cu_3_Br rings based on the “in-plane” Br atoms, the outer scaffold of **2** shows topological analogy to the theoretical structure of *I*-C_140_ fullerene (Figure 2 d). In addition, 30 “out-of-plane” Br atoms bridge the 30 Cu–Cu edges of the outer framework. This results in overall 170 scaffold positions of the outer shell (P_60_Cu_60_Br_50_). In the aggregate **2**, icosahedral symmetry is violated by small distortions caused by the presence of different types of atoms. In contrast, the structural motif of the *I*-C_140_ fullerene has not yet been proven experimentally, although numerous calculations on the structure and physical properties have been performed for the carbon cluster.[9–11, 25] The diameter of the outer scaffold of **2** of roughly 18.0 Å is much larger than the predicted diameter of *I*-C_140_ (10.5 Å; see Ref. [2b]).

The scaffold of the next shell shows a pentagon-dodecahedral shape (*d*_ø_≍14 Å). The nodes of the dodecahedron are occupied by 20 Cu atoms and all edges are bridged in an almost linear fashion (Cu-Br-Cu≍170°) by 30 Br ions (Figure 2 c,e). The outer and middle shells are linked together by numerous Cu–Br bonds (Figure 2 c). Twelve additional CuBr fragments are connected to two adjacent Br ions such that each pentagonal {Cu_5_Br_5_} cycle bears only one these bromides (Figure 2 e). Every Cu ion of this additional CuBr unit coordinates one pentaphosphaferrocene **1** in a η^2^-mode and therefore further links these two shells (Figure 2 f). This also implies that the *cyclo*-P_5_ rings are located directly above the faces of the dodecahedron.

The Br ions of the CuBr fragments point inside the pentagon-dodecahedron. With twelve of these fragments, the Br atoms form a Br_12_ icosahedron as central inner shell. It shows a diameter of about 8.6 Å and an inner cavity of roughly 4.7 Å. An icosahedral core structure is reminiscent of Mackay topology.[26] However, these concepts cannot fully be applied in the present case due to the presence of directed covalent bonds rather than a close packing of spheres. Nevertheless, similar arrangements of multiple-shell clusters can be observed for late transition metal clusters. For instance, Dahl et al. synthesized large nanosized Pd clusters also exhibiting an icosahedral core structure and an outer dodecahedral structure.[27] Another multishell cluster has been reported by Eichhorn et al. who synthesized an As–Ni cluster that also shows the structural feature of an icosahedron interpenetrating a pentagonal dodecahedron.[28]

In general, the whole structure of the aggregate can be described as an icosahedron@pentagon-dodecahedron@“*I*-C_140_”. Interestingly, two of the inner shells are dual to one another, since the vertices of the icosahedron correspond to the faces of the pentagon-dodecahedron (Figure 2 e) and vice versa. Note, this is only the case with full occupation of all atom positions (see the Supporting Information). However, some of the Cu and Br positions in all shells are vacant. X-ray crystallography establishes the cluster as [{Cp^BIG^FeP_5_}_12_Cu_70_Br_83_] and the best description of the crystal structure is a solid solution of different possible isomers. This is a common feature of fullerene-like spheres[20, 29] and is also known for huge coinage-metal chalcogen clusters[30] as well as the polyoxomolybdate giant anions.[31] The different Cu and Br content has also been proven by the elemental analysis. A possible oxidation of the building block **1** could be ruled out by zero-field ^57^Fe Mössbauer measurements, indicating exclusively Fe^II^ centers. The EPR and SQUID measurements confirm that cluster **2** is diamagnetic. This makes the presence of Cu^II^ improbable which could explain the “missing” positive charges. Various NMR investigations also exclude the possibility of a multiply protonated aggregate. (For a detailed description of the performed experiments, the data analyses, and the description of the structure see the Supporting Information.)

It was not possible to detect the molecular ion peak of **2** by mass spectrometry, which is usually not the case for such clusters.[17–19] The ESI mass spectrum (CH_2_Cl_2_/CH_3_CN) of **2** shows peaks at *m*/*z* 2082.0 and 1937.4 for [(Cp^BIG^FeP_5_)_2_Cu_2_Br]^+^ and [(Cp^BIG^FeP_5_)_2_Cu]^+^, respectively, with low intensities. The base peak corresponds to [Cu(CH_3_CN)(CH_2_Cl_2_)]^+^.

The ^31^P{^1^H} NMR spectrum of **2** in a mixture of CH_2_Cl_2_ and CD_3_CN shows one very broad signal at about *δ*=30 ppm. The signal is not well resolved, which on the one hand can be explained by the nonequivalent P atoms and on the other hand by the low solubility. The ^1^H NMR spectrum of **2** displays several superimposed sets of signals, which is consistent with nonrotating Cp^BIG^ ligands. The ^31^P{^1^H} MAS NMR spectrum shows one very broad, but symmetric signal at *δ*=30 ppm with *ω*_1/2_≍5800 Hz.

In summary, the change to the sterically highly demanding Cp^BIG^ ligand resulted in an effective expansion of the aggregate. The synthesized giant spherical cluster **2** has a diameter of about 3.5 nm. It exhibits structural analogy to the hitherto unknown *I*-C_140_ fullerene, a structural motif that that has not yet been observed experimentally. In contrast to the supramolecules obtained from pentaphosphaferrocenes, compound **2** shows a three-shell molecular structure which can strikingly be described as icosahedron@pentagonal-dodecahedron@“*I*-C_140_”. The cluster shows poor solubility, but it could be characterized by NMR spectroscopy. Because of the successful concept of increasing the steric bulk for the formation of larger supramolecules, it remains open where the boundaries of this concept lie.

## Experimental Section

All experiments were carried out under an atmosphere of dry argon or nitrogen using glovebox and Schlenk techniques. Solvents were purified, dried, and degassed prior to use. CuBr was used as obtained by commercial suppliers, [Cp^BIG^Fe(η^5^-P_5_)][21] was prepared according to literature procedures. The NMR spectra in solution were measured on Bruker Avance 300, 400, and 600 spectrometers. The MAS NMR spectrum was recorded on a Bruker Avance 300 spectrometer by using a double-resonance 2.5 mm MAS probe (^31^P: 121.495 MHz). The spectrum was acquired at MAS rotation frequencies up to 20 kHz with a 90° pulse length of about 2.3 μs and relaxation delays of 120 s (^31^P). ESI mass spectra were measured on a ThermoQuest Finnigan TSG 7000 mass spectrometer. The elemental analysis was determined by Mikroanalytisches Labor, Lehrbereich Anorganische Chemie, TU Munich.

Synthesis of **2**: A solution of CuBr (100 mg, 6.7 mmol) in 5 mL CH_3_CN was treated with a solution of [Cp^BIG^Fe(η^5^-P_5_)] (60 mg, 64 μmol) in 5 mL CH_2_Cl_2_ and the reaction mixture was stirred for 30 min resulting in a brown solution. After the solvent was removed in vacuum the residue was triturated with 10 mL CH_2_Cl_2_ and filtered through a cannula into a thin Schlenk tube. The reaction mixture was layered with 20 mL toluene. After complete diffusion black crystals of **2** were obtained (32 mg, 25 % yield for the idealized cluster). Elemental analysis for [{Cp^BIG^FeP_5_}_12_Cu_**70**_Br_**83**_]⋅5 CH_3_CN: calcd: C 35.72, H 3.56, Br 29.44, Cu 19.75, Fe 2.97, N 0.31, P 8.25 %; found: C 35.75, H 3.58, Br 27.4, Cu 19.02, Fe 2.98, N 0.31, P 8.07 %. ESI-MS (CH_3_CN/CH_2_Cl_2_, cation detection mode): *m*/*z*: 2082.0 (1 %, [(Cp^BIG^FeP_5_)_2_Cu_2_Br]^+^), 1937.4 (2 %, [(Cp^BIG^FeP_5_)_2_Cu]^+^), 189.8 (100 %, [Cu(CH_3_CN)(CH_2_Cl_2_)]^+^), 144.9 (30 %, [Cu(CH_3_CN)_2_]^+^).

## Supplementary material

As a service to our authors and readers, this journal provides supporting information supplied by the authors. Such materials are peer reviewed and may be re-organized for online delivery, but are not copy-edited or typeset. Technical support issues arising from supporting information (other than missing files) should be addressed to the authors.
