# Supplementary material for: Giant Spherical Cluster with I-C140 Fullerene Topology
Source: Angew Chem Int Ed Engl. 2015 Sep 28;54(45):13431–5. doi: 10.1002/anie.201505516 (PMC4691335; doi:10.1002/anie.201505516)
Supplement: Supplementary file 1 [file anie0054-13431-SD1.pdf]

Angewandte Chemie
Eine Zeitschrift der Gesellschaft Deutscher Chemiker

Supporting Information

# Giant Spherical Cluster with *I*-$C_{140}$ Fullerene Topology**

*Sebastian Heinl, Eugenia Peresypkina, Jörg Sutter, and Manfred Scheer**

anie_201505516_sm_miscellaneous_information.pdf

# Supplementary Information

**Contents:**

**1. NMR investigations**

**2. Crystallographic details**

**3. Discussion of performed experiments to clarify charge misbalance**

**4. $^{57}$Fe Mössbauer spectra**

**5. References**

## 1. NMR investigations

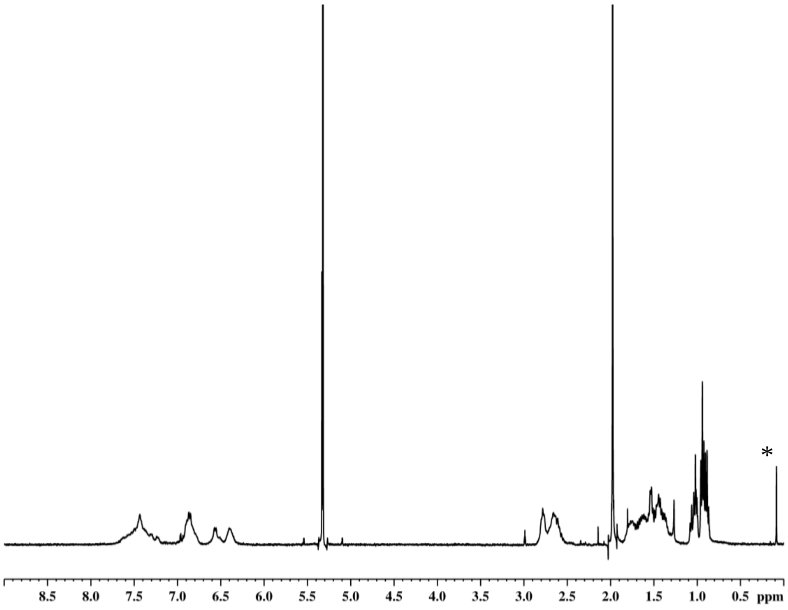


**Figure S1.** $^{1}H$ NMR spectrum of **2** in $CD_2Cl_2$ and $CD_3CN$ at 298 K. Signal marked with an asterisk is due to silicon grease impurities.

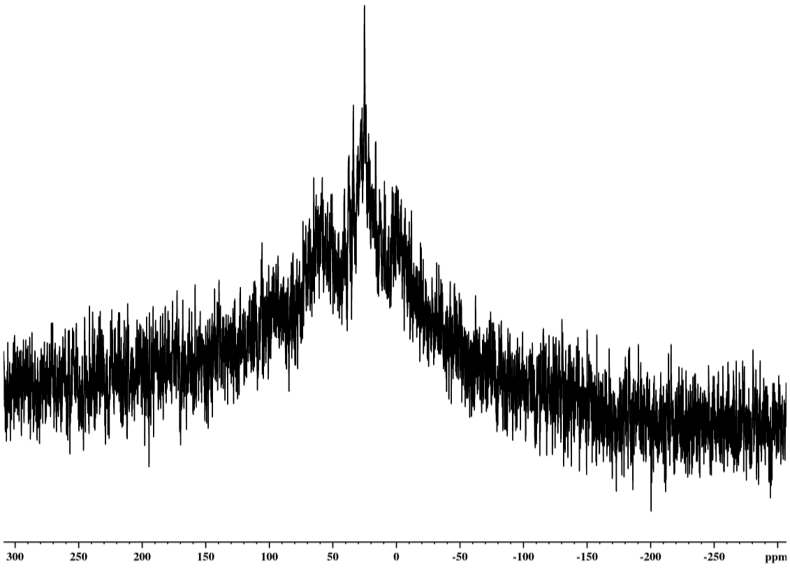


**Figure S2.** $^{31}P\{^{1}H\}$ NMR spectrum of **2** in $CD_2Cl_2$ and $CD_3CN$ at 298 K.

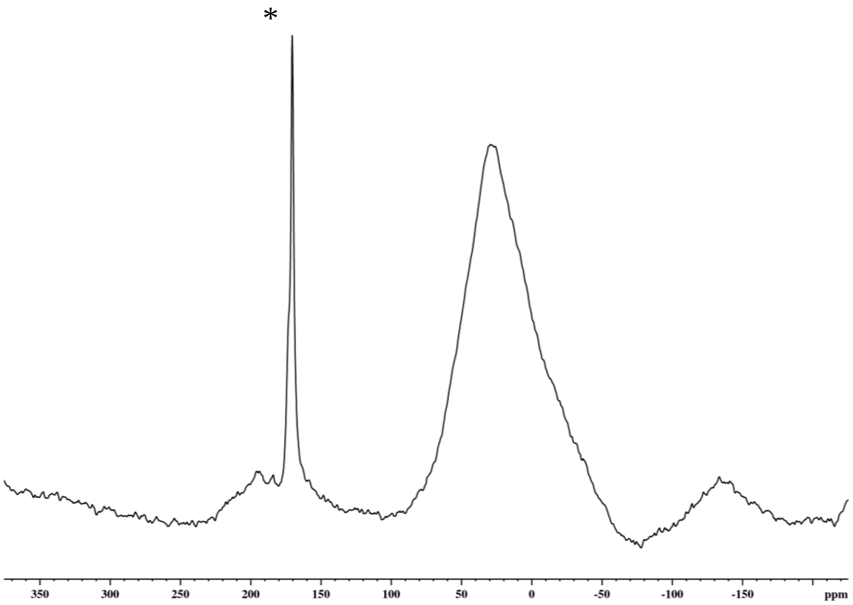


**Figure S3.** $^{31}P\{^{1}H\}$ MAS NMR spectrum of **2** at 298 K. Signal signed with asterisk arises from impurities of **1.**

## 2. Crystallographic details

**Experimental**. Crystals of **2** were taken from a Schlenk tube under a stream of argon in a drop of mother liquor and immediately covered with perfluorinated mineral oil (Fomblin®). The quickly chosen single crystal, covered by a drop of the oil, was taken to the pre-centered goniometer head with CryoMount® and directly attached to the diffractometer into a stream of cold nitrogen.

The crystals showed weak scattering power that rapidly decreased with diffraction angle. This required developing optimal strategy that made possible to collect both weak and strong reflections at different θ angles. For this purpose series of X-ray diffraction experiments of different crystals of **2** obtained from different synthetic approaches was performed on an Agilent

Technologies SuperNova diffractometer equipped with Atlas detector and CuKα SuperNova source using 1° ω scans. All measurements were performed at 123 K. An analytical absorption correction based on crystal faces was carried out.[1] The structure of **2** was solved by the charge-flipping method with the program SUPERFLIP[2] and refined with the least square method on $F^2$ employing multiprocessor and variable memory SHELXL-2014.[3] All heavy atoms were refined in anisotropic approximation as well as the carbon atoms occupying their positions more than 0.5. Hydrogen atoms were placed in idealized positions and refined isotropically according to the riding model. All received experiments were refined with the model elaborated for the first measurement and were further used for comparison. They showed very similar results and the same tendency that some of the Cu and Br positions in the inorganic scaffold are partially vacant indicated by larger displacement parameters. The occupancies for these positions were refined with an average isotropic $U_{iso}$ of 0.05 $Å^{-2}$ typical for fully-occupied heavy atoms in this crystal structure. The resulting occupancies were fixed. The constraints on the Cu and Br displacement parameters were further removed and an anisotropic approximation was used for the further refinement. The best four experiments showed the compositions $Fe_{12}Cu_{69.5(4.1)}Br_{82.7(1.4)}$, $Fe_{12}Cu_{58.3(3.3)}Br_{71.8(1.4)}$, $Fe_{12}Cu_{64.8(4.2)}Br_{76.4(0.2)}$, and $Fe_{12}Cu_{71.4(3.94)}Br_{82.6(1.77)}$.

CCDC-1062705 contain the supplementary crystallographic data for this publication. These data can be obtained free of charge at www.ccdc.cam.ac.uk/conts/retrieving.html (or from the Cambridge Crystallographic Data Centre, 12 Union Road, Cambridge CB2 1EZ, UK; Fax: + 44-1223-336-033; e-mail: deposit@ccdc.cam.ac.uk).

Since all structure refinements lead to the similar results we describe below the structural model received from the best experiment we were able to measure (Table S1). The final structural

formula $[\{C_{55}H_{65}FeP_5\}_{12}Cu_{69.49}Br_{82.70}]\cdot 1.3CH_3CN\cdot 0.34CH_2Cl_2$ does not provide charge balance. The Cu and Br content per one supramolecule are estimated from the refinement with corresponding esds as 69.49±4.01 and 82.70±0.05, respectively. The estimation shows that no charge balance can be achieved within calculating uncertainty. The charge disbalance of about 13 cannot be resolved without an unreasonable increase of the displacement parameters of heavy copper atoms, resulting from an increase of their site occupancy factors. The residual electron density (3.23 eÅ$^{-3}$) and quality factors of the structure refinement ($R_1$ = 0.075; $wR_2$ = 0.264) are low enough to reject the hypothesis that 12-13 cationic copper complexes (e.g. $[Cu(CH_3CN)_4]^+$) are located in the interstitial space between the negatively-charged supramolecular anions.

**Table S1.** Crystallographic data for compound **2**.

| **Crystal data** | |
|---|---|
| Chemical formula | $C_{660}H_{780}Br_{82.70}Cu_{69.45}Fe_{12}P_{60}\cdot(CH_2Cl_2)_{0.34}\cdot(C_2H_3N)_{1.3}$ |
| $M_r$ | 22344.85 |
| Crystal system, space group | Monoclinic, *C*2/*c* |
| Temperature (K) | 123 |
| *a*, *b*, *c* (Å) | 43.1169 (3), 42.1302 (2), 43.2800 (2) |
| β (°) | 92.310 (1) |
| $V$ (Å$^3$) | 78555.2 (8) |
| $Z$ | 4 |
| $F$(000) | 43614 |
| $D_x$ (Mg m$^{-3}$) | 1.889 |
| Radiation type | Cu $K\alpha$ |
| μ (mm$^{-1}$) | 10.09 |
| Crystal shape | Truncated hexagonal prism |
| Colour | Brown |
| Crystal size (mm) | 0.27 × 0.15 × 0.08 |
| **Data collection and refinement** | |
| Diffractometer | SuperNova, Single source at offset, Atlas diffractometer |
| Absorption correction | Gaussian<br>*CrysAlis PRO*, Agilent Technologies, Version 1.171.37.34 (release 22-05-2014 CrysAlis171 .NET) Numerical absorption correction based on gaussian integration over a multifaceted crystal model Empirical absorption correction using spherical harmonics, implemented in SCALE3 ABSPACK scaling algorithm. |
| $T_{min}$, $T_{max}$ | 0.138, 0.533 |
| No. of measured, independent and observed [$I > 2\sigma(I)$] reflections | 427909, 79287, 53189 |
| $R_{int}$ | 0.052 |
| $(\sin \theta/\lambda)_{max}$ (Å$^{-1}$) | 0.627 |
| Range of *h*, *k*, *l* | $h$ = -53→52, $k$ = -52→52, $l$ = -44→53 |
| $R[F^2 > 2\sigma(F^2)]$, $wR(F^2)$, $S$ | 0.075, 0.264, 1.06 |
| No. of reflections | 79287 |
| No. of parameters | 4481 |
| No. of restraints | 66 |
| H-atom treatment | H-atom parameters constrained |
| $\Delta\rangle_{max}$, $\Delta\rangle_{min}$ (e Å$^{-3}$) | 3.23, -2.45 |

The *n*-butyl (*n*Bu) groups of the pentaphosphaferrocene ligands are often disordered over two, sometimes three positions. The occupancies of the positions were refined with $U_{iso}$ = 0.08 Å$^{-2}$, allowing higher displacements for light atoms and were then fixed in the obtained values. The carbon atoms of *n*Bu groups are disordered in many cases and were refined isotropically when the occupancies were less than 0.5. The phenyl groups are sometimes disordered, too. The disordered light atoms were refined with a number of geometrical restraints. Some disordered *n*Bu groups were refined so that subsequent C atom riding on the preceding C atom with U (subsequent) = 1.1U (preceding) or 1.2 U (preceding) depending on the previously refined $U_{iso}$ magnitudes.  Due to high portion of heavy atoms in the structure the minor positions of the disordered ligands could not be localized. In this case the major position was treated as fully occupied that caused high displacement parameters for the carbon atoms. This also led to a number of contradictory short contacts H…H ~ 1.6-1.7 Å.

The maximal peak of the electron density is 3.23 eÅ$^{-3}$ is located in the geometrical center of the supramolecule (positioned on the 2-fold axis) and most probably corresponds to either highly disordered solvent molecule or isolated Br anion. In the latter case the contribution of the negative charge to the sum formula would be negligible. The peak is therefore left unassigned. Other peaks of 2.39 eÅ$^{-3}$ or less are mainly located in the close proximity of heavy atoms.

Only minor content of solvent $CH_2Cl_2$ and acetonitrile molecules was localized. All solvent molecules partially occupy their positions. Therefore, they could only be refined with geometrical restraints. According to the PLATON (SQUEEZE)[4] the solvent accessible voids of total 2145.9 Å$^3$ contain 405 electrons per unit cell (536.5 Å$^3$ and 101.25 electrons per supramolecule, Z = 4). Nevertheless, largest voids of 172 Å$^3$ contain about 34-41 electrons that

may correspond to one DCM or two acetonitrile molecules more. The central cavity of 85 Å$^3$ comprises only 10 electrons and is the least trustworthy value due to severe disorder of the inner surface of the cavity. These values should be treated with care since no reliable estimation can be done for essentially disordered structures.

**Description of the vacant scaffold.** In the outer shell all Br positions are fully occupied, while 8.5 Cu ions in total are statistically missing with a different probability (0.0 – 0.3) in every position. In addition, every position of Cu is split into two close ones. In the pentagon-dodecahedron of the inner shell (Figure 2c), only 7.06 Cu ions are disordered over 20 available positions, but all Br positions are fully occupied. The occupancies of atoms in the CuBr fragments linking pentagon-dodecahedron with inner icosahedral shell (Figure 2e,f) are much higher for Cu than for Br. Therefore, the 12 CuBr fragments which appear in idealized structure are reduced to 2 or 3 per supramolecule (2.7 in average) and 9 or 8 Cu ions with one vacant coordination site respectively. In total, only 11 of 12 pentaphosphaferrocenes are coordinated to the Cu ions in $\eta^2$-mode.

Inexplicably, the structure of the supramolecule is favorable to a complete saturation of all the positions of Cu and also Br, but it never takes place. Even the 100-fold excess of CuBr in the synthesis did not lead to saturation of the partly occupied positions of copper in the scaffold and neutral composition $[\{Cp^{BIG}FeP_5\}_{12}Cu_{92}Br_{92}]$ was never achieved. The maximal saturation we could reach was $[\{Cp^{BIG}FeP_5\}_{12}Cu_{71.4}Br_{82.6}]$. The reason for the 'missing' charge was therefore thoroughly investigated with other methods.

Analysis of crystal packing of supramolecules was performed with TOPOSPro software.[5] The supramolecules are arranged in a crystal following a slightly distorted motif of the face-centered cubic (f.c.c.) packing (Figure S4).

In conclusion, taking into account the variable CuBr-composition in the series of crystals and the disorder in the inorganic scaffold in each structure we can therefore state that compound **2** should be considered as a solid solution of isomers. The isomers differ by the amount and location of vacancies in the idealized molecular structure described above. One of the isomers with non-contradictory molecular structure is shown in Figure S5b.

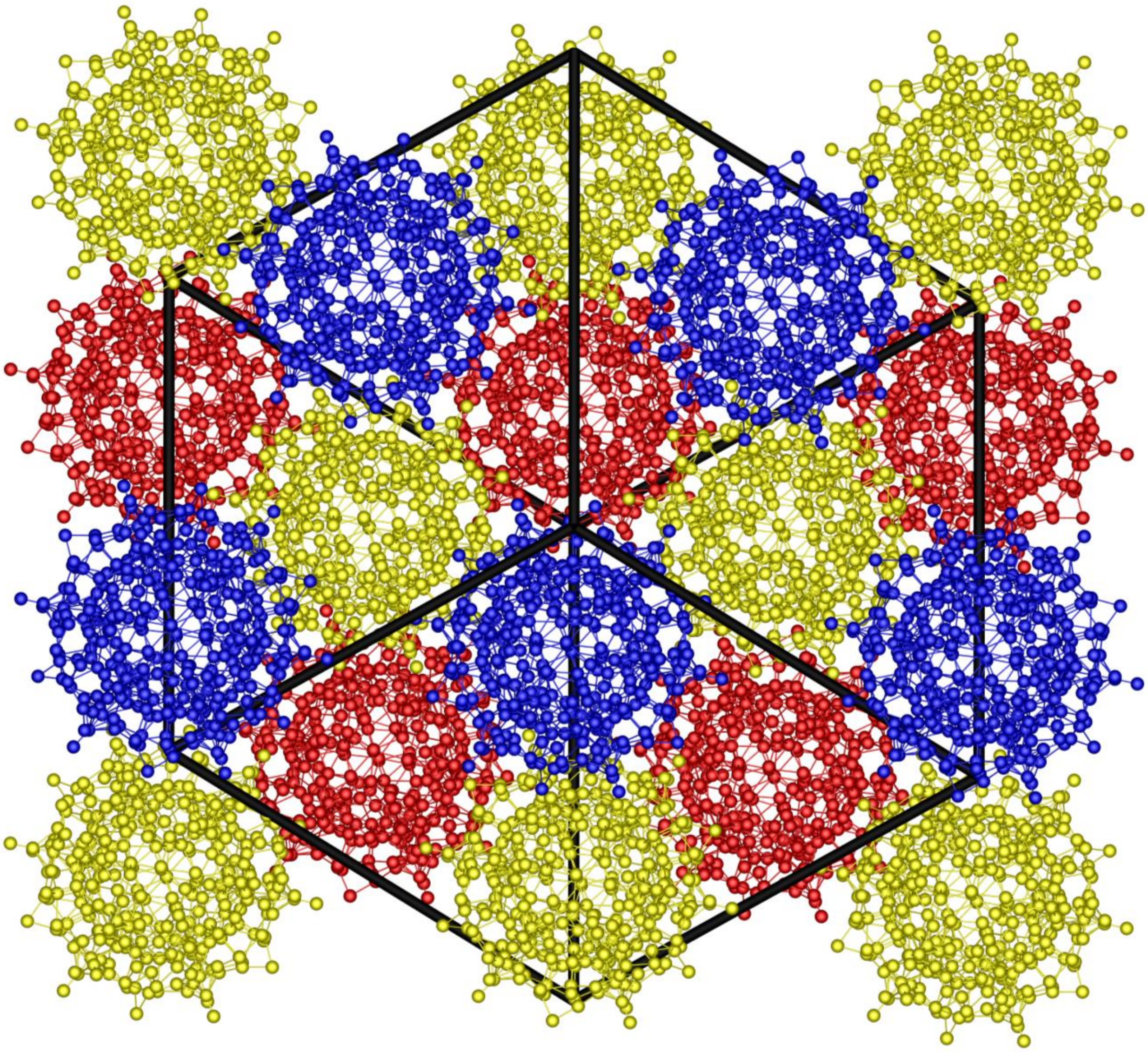

**Figure S4.** Three-layered face-centered cubic close packing of aggregates **2** (inorganic scaffold only) in the crystal, view along the [111] direction. Each layer is drawn in a different color.

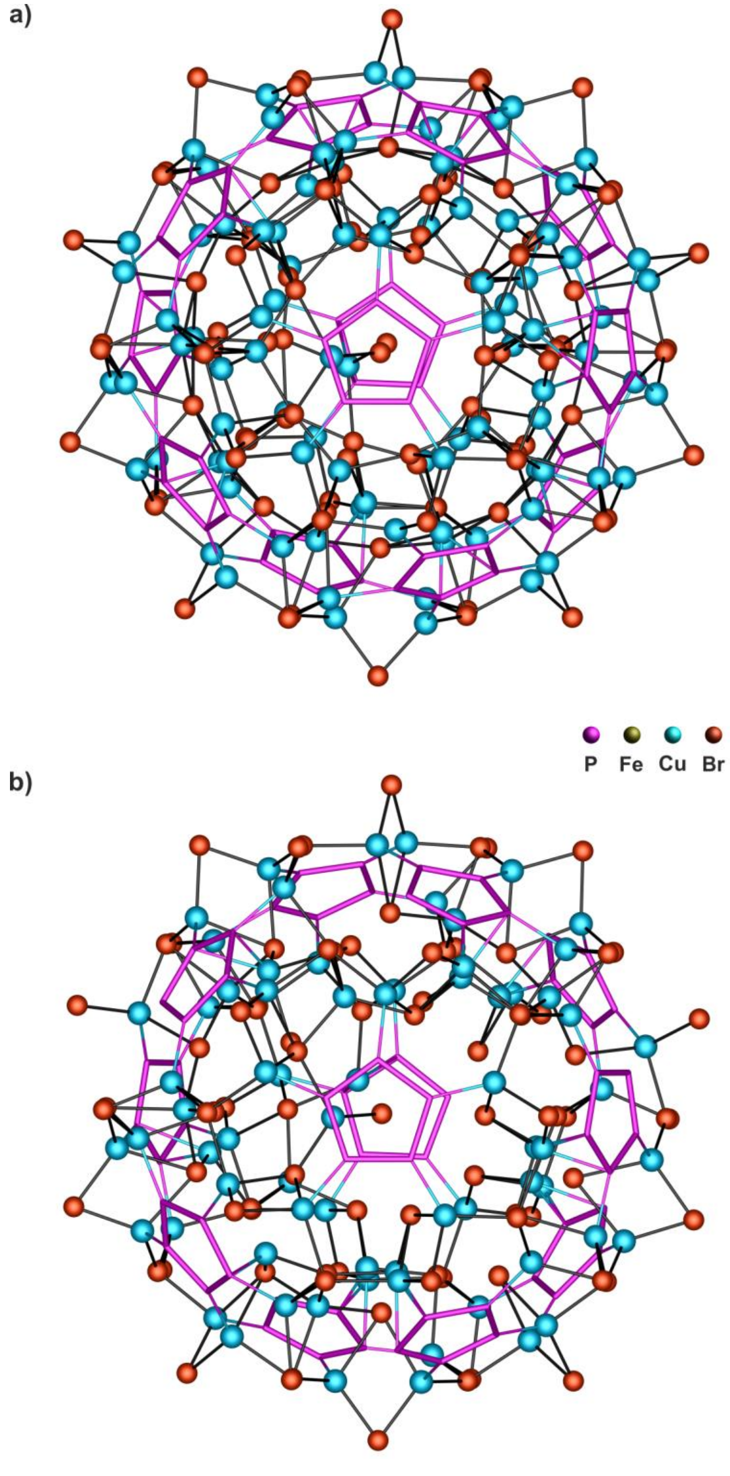


**Figure S5.** Inorganic scaffolds of **2**. **a)** Idealized aggregate $Cu_{92}Br_{92}P_{60}$ with fully occupied heavy atom positions. **b)** The partly vacant inorganic scaffold of one possible isomer of $Cu_{70}Br_{83}P_{60}$ with most probable vacancies.

## 3. Discussion of the performed experiments to clarify charge misbalance

Note that the objectively received difference between Cu and Br is close to 12 and therefore the lack of positive charges could be attributed to one-electron oxidation of the pentaphosphaferrocenes **1**. We measured zero field $^{57}$Fe Mößbauer spectra of compound **1** and **2** to verify, if the 'missing' positive charges are located on the iron atoms of the building blocks **1**. In both spectra almost identical isomer shifts and quadrupole splittings are observed. Furthermore, the characteristics indicate the presence of Fe(II) centers rather than of Fe(III). It is also possible that the charges are located on either the $P_5$-ring or the $Cp^{BIG}$ ligands, resulting in radical cations. However, EPR studies of compound **2** in solution and solid state at r.t. and 77 K did not show any signal indicating radicals. Though, the unpaired electrons could be antiferromagnetically coupled or do show very fast relaxation. In addition, it is possible to have Cu(I) and Cu(II) mixed in the same position. To get more insight into this, SQUID measurements were recorded. The found diamagnetism of the cluster in the whole temperature range (2 K – 300 K) makes the presence of Cu(II) cations improbable. Nevertheless, a strong antiferromagnetic coupling could not be excluded.

Another possible explanation for the positive charges could be protonation of solvent molecules, allocated in the interspaces between the clusters, or the cluster itself, respectively. To check this, two portions of crystals were prepared, one from $^{1}$H containing solvents the other by using only deuterated solvents during the whole synthesis. Then one half each of the isolated crystals were dissolved in pyridine and pyridine-$d_5$, respectively. This leads to the complete destruction of the aggregate. If protons or deuterons are present, the protonated or deuterated pyridinium cations, respectively, should be formed. However, in all four samples no appropriate signals in the $^{1}$H and $^{2}$H NMR spectra for pyridinium cations could be found. Hence the aggregate is not protonated.

Unfortunately, a final statement on the ‘missing’ positive charges cannot be made.

## 4. $^{57}$Fe Mößbauer spectra

Zero Field $^{57}$Fe Mößbauer spectra were recorded on a WissEl Mößbauer spectrometer (MRG-500) at 77 K in constant acceleration mode. $^{57}$Co/Rh was used as the radiation source. WinNormos for Igor Pro software has been used for the quantitative evaluation of the spectral parameters (least squares fitting to Lorentzian peaks). The minimum experimental line widths were 0.20 $mms^{-1}$. The temperature of the samples was controlled by an MBBC-HE0106 MÖßBAUER He/$N_2$ cryostat within an accuracy of ±0.3 K. Isomer shifts were determined relative to α-iron at 298 K.

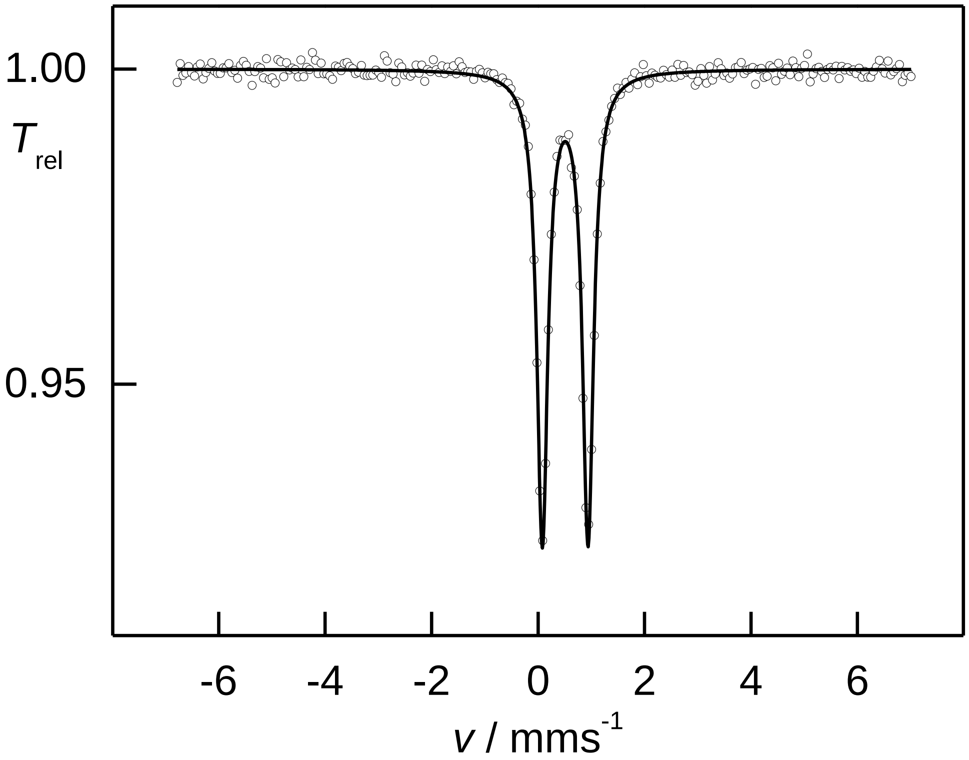


**Figure S6.** $^{57}$Fe Mößbauer spectrum of compound **1**.

Spectrum:

Type: doublet

$\delta$ = 0.51(1) mms$^{-1}$

$\Delta E_Q$ = 0.86(1) mms$^{-1}$

$\Gamma_{FWHM}$ = 0.25(1) mms$^{-1}$

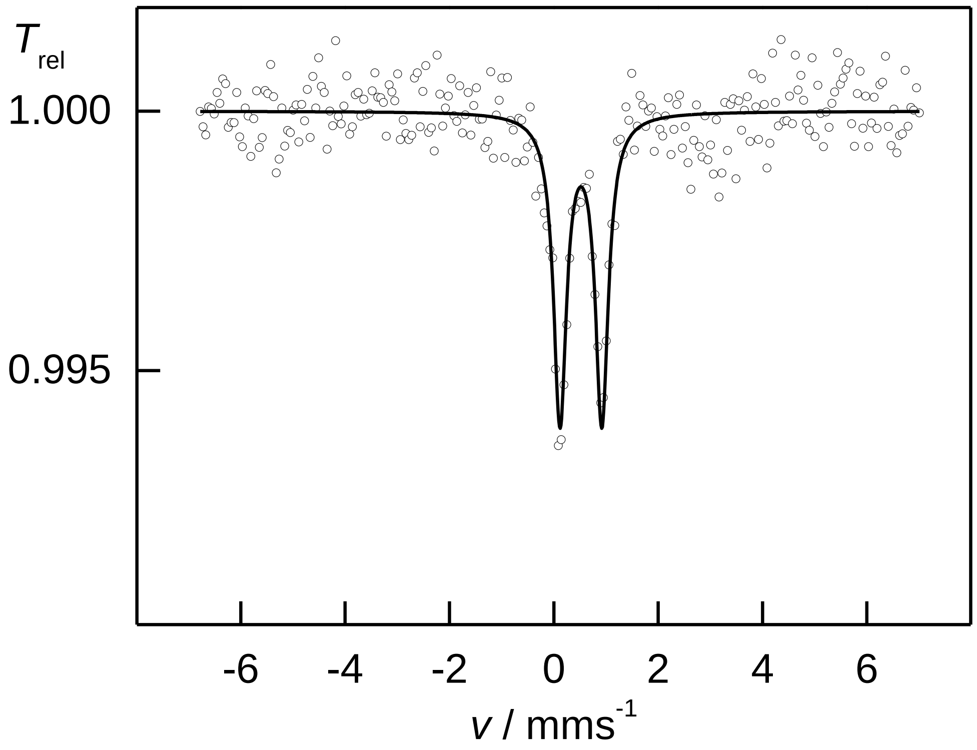


**Figure S7.** $^{57}$Fe Mößbauer spectrum of compound **2**.

Spectrum:

Type: doublet

$\delta$ = 0.52(1) mms$^{-1}$

$\Delta E_Q$ = 0.80(1) mms$^{-1}$

$\Gamma_{FWHM}$ = 0.30(1) mms$^{-1}$

## 5. References


1. R. C. Clark, J. S. Reid, *Acta Cryst. A* **1995**, *51*, 887-897.
2. L. Palatinus, G. Chapuis, *J. Appl. Crystallogr.* **2007**, *40*, 786-790.
3. G.M. Sheldrick, *Acta Cryst.* **2015**, *C71*, 3.
4. P. van der Sluis, A. L. Spek, *Acta Cryst. A* **1990**, *46*, 194-201.
5. V. A. Blatov, A.P. Shevchenko, D. M. Proserpio, *Cryst. Growth Des.* **2014**, *14*, 3576.
